# Supplementary material for: Regional, institutional, and departmental factors associated with gender diversity among BS-level chemical and electrical engineering graduates
Source: PLoS One. 2019 Oct 9;14(10):e0223568. doi: 10.1371/journal.pone.0223568 (PMC6785119; doi:10.1371/journal.pone.0223568)
Supplement: S1 Table — (PDF) [file pone.0223568.s001.pdf]

S1 Table: Institution Data

	IPEDS																IPEDS													
Institution Name	2010 ChE total	2010 EE total	2011 ChE total	2011 EE total	2012 ChE total	2012 EE total	2013 ChE total	2013 EE total	2014 ChE total	2014 EE total	2015 ChE total	2015 EE total	2016 ChE total	2016 EE total	# total ChE BS, 2010 - 2016	# total EE BS, 2010 - 2016	2010 ChE female	2010 EE female	2011 ChE female	2011 EE female	2012 ChE female	2012 EE female	2013 ChE female	2013 EE female	2014 ChE female	2014 EE female	2015 ChE female	2015 EE female	2016 ChE female	2016 EE female
Arizona State University-Tempe	43	90	41	99	71	99	71	96	77	110	99	164	166	190	568	848	13	10	20	15	25	11	20	7	15	16	24	13	55	25
Brigham Young University-Provo	49	62	57	61	80	67	88	74	70	73	91	87	86	57	521	481	6	4	5	2	13	4	16	2	8	4	17	4	12	5
California Institute of Technology	18	21	15	25	24	18	31	25	15	16	14	17	14	20	131	142	8	5	6	4	15	5	19	7	6	2	8	3	6	5
California State Polytechnic University-Pomona	37	145	22	141	44	144	43	187	52	195	61	186	47	170	306	1168	13	10	6	14	13	10	19	17	15	11	24	14	22	24
California State University-Long Beach	15	52	29	48	28	60	39	55	45	72	47	85	61	90	264	462	6	4	12	6	9	5	10	6	15	10	16	14	24	7
Carnegie Mellon University	71	182	61	131	64	147	71	124	73	136	73	149	53	149	466	1018	30	32	33	28	19	26	37	17	40	22	37	29	31	37
Case Western Reserve University	31	31	52	27	42	30	33	30	34	38	30	27	54	31	276	214	13	2	15	4	7	3	13	8	18	7	11	4	17	8
Clemson University	35	69	49	52	40	54	36	82	43	77	51	86	52	93	306	513	8	13	9	9	9	5	8	9	14	12	12	11	15	11
Colorado State University-Fort Collins	28	23	32	15	51	30	42	28	59	37	34	47	49	42	295	222	9	1	12	2	13	2	13	5	17	2	14	8	18	4
Cooper Union for the Advancement of Science and Art	19	31	27	36	24	28	20	29	27	30	21	27	19	28	157	209	9	6	11	4	7	8	11	5	7	7	6	4	10	1
Cornell University	85	100	71	74	72	88	98	69	82	64	81	78	100	93	589	566	33	17	26	8	35	10	44	10	39	8	39	15	46	30
Drexel University	51	68	63	74	65	66	51	72	56	81	76	97	94	116	456	574	22	6	20	12	27	7	19	7	28	6	32	15	32	15
Florida State University	34	23	14	35	41	33	10	47	30	59	64	50	66	47	259	294	15	2	5	3	15	3	2	8	9	8	18	10	22	3
Georgia Institute of Technology-Main Campus	100	220	128	200	142	203	158	238	165	233	172	239	189	242	1054	1575	46	19	42	20	39	22	58	22	49	33	60	25	62	35
Hampton University	3	12	3	0	4	7	4	12	6	21	5	0	7	0	32	52	3	4	2	0	0	0	4	5	3	6	2		3	
Howard University	8	14	10	12	17	10	23	14	16	15	20	12	13	10	107	87	3	5	6	3	14	1	15	1	9	4	15	6	6	1
Iowa State University	63	73	94	76	78	67	113	104	103	105	108	128	159	108	718	661	18	3	43	5	26	4	38	14	51	13	37	12	48	10
Kansas State University	26	43	28	34	35	38	21	36	45	54	31	42	48	50	234	297	8	4	7	3	8	3	5	3	11	3	11	2	13	7
Louisiana State University and Agricultural & Mechanical Colleg	54	70	66	58	65	60	66	62	75	48	77	54	103	56	506	408	15	3	23	5	25	3	18	3	36	3	23	9	40	6
Louisiana Tech University	25	32	18	34	25	27	23	32	25	31	24	24	35	15	175	195	5	3	3	3	7	6	5	3	7	2	9	6	5	2
Massachusetts Institute of Technology	76	67	68	57	75	66	67	68	64	60	65	49	67	52	482	419	41	27	45	14	42	21	41	17	33	15	29	20	40	14
Michigan Technological University	57	104	66	85	73	95	71	87	75	106	78	104	102	81	522	662	15	9	16	10	20	13	25	7	24	9	14	11	37	10
Mississippi State University	39	30	41	38	52	28	48	36	39	39	59	39	75	54	353	264	11	4	12	3	10	3	7	4	7	5	12	4	23	7
Missouri University of Science and Technology	60	83	53	79	57	82	58	90	73	87	73	85	103	101	477	607	18	6	24	5	20	4	23	12	23	10	21	6	36	11
Montana State University	27	23	47	30	72	19	52	26	104	26	136	41	77	31	515	196	8	2	21	5	34	3	21	2	49	2	43	3	33	5
New Mexico Institute of Mining and Technology	11	22	16	19	14	25	16	11	23	16	17	17	23	13	120	123	2	3	5	0	6	2	5	2	8	3	9	3	7	3
New Mexico State University-Main Campus	10	37	17	25	17	33	20	22	22	67	27	40	38	43	151	267	4	9	6	5	9	5	9	5	9	11	13	0	16	4
North Carolina A & T State University	14	40	16	36	12	37	9	33	16	20	15	24	22	33	104	223	8	4	10	11	8	11	3	5	10	4	10	1	10	4
North Carolina State University at Raleigh	107	131	123	149	149	145	146	150	158	158	175	176	177	209	1035	1118	35	9	36	15	44	12	37	12	54	19	46	17	64	18
Northwestern University	30	28	30	22	25	19	43	18	39	18	42	12	39	13	248	130	13	4	9	4	11	5	11	4	11	3	11	1	15	7
Oklahoma State University-Main Campus	44	31	40	29	30	29	43	32	43	43	36	42	62	45	298	251	16	4	12	2	16	5	13	4	14	2	9	4	17	3
Oregon State University	41	66	49	66	60	97	71	78	98	90	88	107	88	115	495	619	11	8	19	10	15	3	13	4	25	8	21	17	27	10
Pennsylvania State University-Main Campus	107	141	121	150	121	160	147	157	139	174	147	178	160	190	942	1150	30	10	25	6	31	18	37	14	41	20	38	20	39	19
Prairie View A & M University	17	33	25	26	42	20	56	23	37	27	60	38	62	28	299	195	6	9	14	4	23	11	16	2	11	3	22	7	25	6
Princeton University	36	31	27	22	34	22	44	19	33	10	42	35	45	35	261	174	17	9	14	5	19	7	24	7	18	4	24	11	24	17
Purdue University-Main Campus	142	192	137	172	163	167	138	184	140	145	158	202	146	179	1024	1241	41	16	46	18	52	16	40	25	45	18	49	20	50	31
Rensselaer Polytechnic Institute	51	100	66	77	67	70	73	87	66	70	74	84	82	81	479	569	22	11	26	20	16	13	26	7	25	15	21	10	25	19
Rose-Hulman Institute of Technology	73	40	52	40	51	32	43	63	66	49	72	47	64	52	421	323	27	3	14	5	13	2	16	4	22	5	31	8	19	8
Rowan University	23	19	20	21	22	30	34	25	26	27	41	48	39	34	205	204	9	2	3	1	6	1	11	4	9	3	11	5	9	2
Rutgers University-New Brunswick	56	102	59	112	69	148	71	139	70	129	99	152	104	153	528	935	23	10	17	8	19	13	17	17	24	10	33	19	36	20
San Jose State University	19	95	12	72	18	77	24	72	26	71	25	90	53	100	177	577	7	8	4	11	5	12	6	9	9	6	5	12	20	5
South Dakota School of Mines and Technology	15	23	25	13	18	23	27	26	32	18	36	20	32	20	185	143	4	0	7	1	5	2	7	3	12	0	13	4	14	3
Stanford University	20	36	23	43	23	39	22	36	22	33	39	42	30	50	179	279	5	7	9	6	12	8	8	9	6	6	14	7	7	5
Texas A & M University-College Station	134	88	113	102	152	108	140	132	154	145	106	154	114	196	913	925	38	14	36	14	44	15	41	13	48	18	34	23	28	32
The Johns Hopkins University	81	14	70	17	58	17	65	25	62	17	76	24	54	30	466	144	39	0	17	3	23	6	23	2	21	7	24	5	20	8
The Ohio State University-Main Campus	96	149	115	125	129	169	118	171	176	203	202	215	180	205	1016	1237	23	9	24	8	24	19	34	11	50	25	61	31	48	22
The University of Alabama	42	46	44	46	58	53	63	60	88	52	106	56	172	92	573	405	9	4	19	7	18	6	18	11	37	7	34	10	64	20
The University of Kansas	46	36	42	25	56	28	52	34	51	35	55	26	71	40	373	224	24	3	13	2	19	6	19	4	18	3	20	3	20	2
The University of Tennessee-Knoxville	28	49	28	41	37	56	34	44	49	56	63	44	58	50	297	340	7	2	11	1	12	7	9	3	24	4	14	3	13	9
The University of Texas at Austin	124	216	132	245	128	236	157	232	137	269	171	255	152	264	1001	1717	40	31	48	26	40	26	58	30	60	47	58	30	51	34
Tuskegee University	13	15	13	21	6	17	14	11	15	13	15	19	15	20	91	116	12	3	7	7	4	4	6	2	9	3	8	7	11	4
University of Arkansas	31	36	43	36	29	29	36	41	66	60	48	43	63	49	316	294	9	6	12	3	7	3	12	4	19	5	18	5	30	4
University of California-Berkeley	83	268	86	260	89	311	80	330	124	407	108	367	103	365	673	2308	32	25	24	21	29	35	25	39	38	42	33	51	23	41
University of California-Davis	54	2	57	1	70	6	69	4	92	0	95	2	125	0	562	15	17	1	17	1	30	0	19	0	33	0	23	0	41	0
University of California-Los Angeles	84	128	102	173	85	172	85	153	90	151	78	156	82	125	606	1058	36	22	34	21	26	25	27	23	28	22	20	27	28	20
University of California-San Diego	52	95	66	99	51	108	76	112	113	114	134	132	193	215	685	875	20	16	26	13	10	17	26	17	31	12	47	20	70	19
University of California-Santa Barbara	40	45	40	40	47	52	63	41	47	57	51	49	54	64	342	348	13	5	12	6	10	3	15	4	17	4	13	4	12	9
University of Colorado Boulder	55	29	54	23	52	39	39	38	43	38	45	48	81	20	369	235	26	3	16	0	11	6	9	5	17	6	18	7	25	0
University of Connecticut	23	35	47	36	46	36	52	44	50	58	41	55	67	61	326	325	8	2	17	2	10	2	17	2	8	9	15	10	21	11
University of Delaware	34	28	49	31	65	25	52	34	76	45	87	36	85	41	448	240	9	2	15	2	15	2	16	6	23	9	17	2	16	7
University of Florida	74	87	82	119	87	114	103	82	99	110	132	111	143	99	720	722	22	11	29	10	28	12	30	13	28	22	42	18	51	9
University of Idaho	17	31	13	14	14	26	19	22	29	32	17	24	22	39	131	188	6	3	6	2	4	4	2	3	9	4	5	0	8	1
University of Illinois at Urbana-Champaign	78	202	110	177	105	232	97	241	120	295	159	266	122	250	791	1663	25	18	30	20	31	33	33	31	38	44	52	31	34	30
University of Iowa	24	31	39	38	31	36	25	45	26	68	27	87	44	76	216	381	5	0	18	4	9	4	6	5	7	10	4	13	12	16
University of Kentucky	35	48	35	50	35	45	42	58	47	63	78	53	62	74	334	391	11	6	11	6	13	3	12	5	15	4	23	4	26	10
University of Louisville	35	48	23	51	30	50	31	54	28	58	35	48	39	46	221	355	13	5	4	4	8	1	9	6	6	7	12	8	12	2
University of Maine	16	23	23	27	28	17	32	23	21	13	29	13	40	24	189	140	3	0	8	1	7	0	6	2	5	3	9	0	11	1
University of Maryland-College Park	44	93	58	118	53	130	61	128	72	140	66	120	76	125	430	854	16	13	20	26	18	9	23	10	24	20	15	21	26	18
University of Massachusetts-Amherst	45	42	41	36	70	46	80	28	69	51	85	50	108	54	498	307	15	1	14	3	20	7	15	5	18	6	25	8	19	6
University of Michigan-Ann Arbor	128	92	142	108	146	126	132	123	135	128	144	113	173	104	1000	794	39	13	47	18	47	23	43	22	51	25	51	24	56	21
University of Minnesota-Duluth	26	40	26	23	40	21	26	42	38	33	55	33	44	44	255	236	7	4	4	0	11	1	3	5	6	0	10	2	15	6
University of Minnesota-Twin Cities	100	123	98	122	121	125	106	140	128	113	95	138	114	126	762	887	35	7	21	8	24	21	24	12	32	17	21	17	32	11
University of Mississippi	9	14	14	14	17	7	16	6	19	15	38	10	31	13	144	79	3	3	7	2	6	2	7	1	11	4	11	1	9	0
University of Nebraska-Lincoln	17	50	17	59	28	46	29	57	29	67	29	57	32	67	181	403	6	6	1	2	6	2	6	5	8	11	6	5	7	3
University of Nevada-Reno	20	23	14	25	10	22	15	25	22	29	23	45	22	36	126	205	6	4	3	4	2	5	6	4	7	1	5	8	5	4
University of New Hampshire-Main Campus	18	20	33	35	32	24	31	33	48	29	36	33	32	25	230	199	5	1	9	2	7	4	8	4	15	2	8	2	10	2
University of North Dakota	31	25	28	28	14	32	23	36	30	37	36	44	37	57	199	259	5	3	3	2	4	2	3	0	6	1	6	6	11	8
University of Oklahoma-Norman Campus	42	26	39	26	55	31	73	44	60	41	51	61	92	53	412	282	16	1	13	3	21	5	27	4	18	6	18	7	36	8
University of Pennsylvania	49	31	34	24	45	26	46	29	35	25	34	18	42	16	285	169	17	1	15	5	15	5	16	3	19	8	16	5	11	4
University of Pittsburgh-Pittsburgh Campus	50	50	54	55	75	57	74	52	99	55	97	53	108	57	557	379	14	5	13	8	34	3	20	7	23	7	28	7	39	9
University of Puerto Rico-Mayaguez	84	111	90	82	115	85	116	93	87	75	69	78	86	81	647	605	60	22	56	18	68	16	68	15	44	9	37	20	46	16
University of Rhode Island	20	35	31	36	23	23	33	27	27	22	25	33	32	35	191	211	6	5	13	4	10	2	11	4	7	0	6	3	10	1
University of South Carolina-Columbia	27	32	31	42	34	30	39	29	36	25	39	49	48	36	254	243	7	4	9	0	7	5	12	2	12	0	15	9	16	4
University of Utah	26	41	30	46	30	53	54	66	57	47	63	57	55	52	315	362	8	8	5	1	4	2	7	4	12	4	12	3	14	7
University of Washington-Seattle Campus	60	164	54	156	63	161	63	148	71	178	80	190	75	184	466	1181	26	19	20	27	20	23	18	17	26	43	28	44	20	40
University of Wisconsin-Madison	49	60	71	65	85	60	95	63	89	62	76	79	96	68	561	457	15	6	19	9	14	2	27	4	19	9	20	10	31	13
University of Wyoming	21	16	16	23	24	26	37	18	29	21	28	13	34	16	189	133	5	1	4	1	6	3	7	1	4	2	10	0	8	1
Vanderbilt University	29	28	44	20	49	22	47	16	57	26	57	28	49	27	332	167	13	10	22	4	25	7	23	4	23	7	29	6	17	6
Virginia Polytechnic Institute and State University	57	103	105	100	79	90	103	107	96	144	107	128	92	120	639	792	17	9	26	14	33	10	36	9	17	19	36	11	38	14
Washington State University	17	60	31	51	19	67	35	77	30	92	52	107	56	96	240	550	4	8	6	3	7	4	8	9	10	2	16	12	10	11
Washington University in St Louis	29	9	31	12	32	18	34	28	36	28	60	39	59	38	281	172	12	1	8	0	7	4	11	8	11	9	26	7	19	4
West Virginia University	10	41	30	20	33	40	42	35	37	43	43	43	59	50	254	272	4	4	8	4	10	3	14	5	11	3	12	6	19	6
West Virginia University Institute of Technology	2	7	7	12	11	9	1	11	5	7	9	10	4	12	39	68	0	1	3	0	5	2	0	0	1	0	4	2	1	2
Worcester Polytechnic Institute	57	78	46	74	73	91	69	86	65	98	104	97	84	103	498	627	21	8	19	7	29	16	28	12	34	21	47	17	36	22
Yale University	5	6	1	6	11	2	8	3	7	2	10	5	14	4	56	28	2	0	1	0	4	0	3	1	2	1	3	3	7	0
sum of focal	4241	6004	4655	5830	5151	6191	5396	6479	5825	6970	6352	7285	7000	7463																
ASEE	5948	9634	6487	9942	7245	10102	7717	10662	8110	11261	9090	11385	9864	11802																
NSF	6692	16290	7535	16485	8344	17380	8917	18412	9706	19991	10837	21357																		

S1 Table: Institution Data

Institution Name	2010 ChE % female	2011 ChE % female	2012 ChE % female	2013 ChE % female	2014 ChE % female	2015 ChE % female	2016 ChE % female	ChE slope	ChE slope standard deviation	ChE p value, non-zero slope	2010 EE % female	2011 EE % female	2012 EE % female	2013 EE % female	2014 EE % female	2015 EE % female	2016 EE % female	EE slope	EE slope standard deviation	EE p value, non-zero slope	ChE BS bulk % female 2010 - 2016	EE BS bulk % female 2010 - 2016	ChE PhD?	EE PhD?
Arizona State University-Tempe	30.23	48.78	35.21	28.17	19.48	24.24	33.13	-2.004	1.716	0.300	11.11	15.15	11.11	7.29	14.55	7.93	13.16	-0.174	0.628	0.790	30.28	11.44	y	y
Brigham Young University-Provo	12.24	8.77	16.25	18.18	11.43	18.68	13.95	0.719	0.690	0.300	6.45	3.28	5.97	2.70	5.48	4.60	8.77	0.325	0.398	0.450	14.78	5.20	y	y
California Institute of Technology	44.44	40.00	62.50	61.29	40.00	57.14	42.86	0.251	2.090	0.900	23.81	16.00	27.78	28.00	12.50	17.65	25.00	-0.300	1.260	0.820	51.91	21.83	y	y
California State Polytechnic University-Pomona	35.14	27.27	29.55	44.19	28.85	39.34	46.81	2.088	1.316	0.170	6.90	9.93	6.94	9.09	5.64	7.53	14.12	0.556	0.530	0.340	36.60	8.56	n	n
California State University-Long Beach	40.00	41.38	32.14	25.64	33.33	34.04	39.34	-0.552	1.120	0.640	7.69	12.50	8.33	10.91	13.89	16.47	7.78	0.491	0.666	0.490	34.85	11.26	n	n
Carnegie Mellon University	42.25	54.10	29.69	52.11	54.79	50.68	58.49	2.393	1.730	0.230	17.58	21.37	17.69	13.71	16.18	19.46	24.83	0.586	0.703	0.440	48.71	18.76	y	y
Case Western Reserve University	41.94	28.85	16.67	39.39	52.94	36.67	31.48	0.734	2.330	0.760	6.45	14.81	10.00	26.67	18.42	14.81	25.81	2.374	1.148	0.090	34.06	16.82	y	y
Clemson University	22.86	18.37	22.50	22.22	32.56	23.53	28.85	1.370	0.764	0.130	18.84	17.31	9.26	10.98	15.58	12.79	11.83	-0.848	0.623	0.230	24.51	13.65	y	y
Colorado State University-Fort Collins	32.14	37.50	25.49	30.95	28.81	41.18	36.73	0.873	1.063	0.450	4.35	13.33	6.67	17.86	5.41	17.02	9.52	0.773	1.093	0.510	32.54	10.81	y	y
Cooper Union for the Advancement of Science and Art	47.37	40.74	29.17	55.00	25.93	28.57	52.63	-0.421	2.510	0.870	19.35	11.11	28.57	17.24	23.33	14.81	3.57	-1.614	1.527	0.340	38.85	16.75	n	n
Cornell University	38.82	36.62	48.61	44.90	47.56	48.15	46.00	1.555	0.700	0.076	17.00	10.81	11.36	14.49	12.50	19.23	32.26	2.277	1.162	0.100	44.48	17.31	y	y
Drexel University	43.14	31.75	41.54	37.25	50.00	42.11	34.04	0.068	1.280	0.960	8.82	16.22	10.61	9.72	7.41	15.46	12.93	0.272	0.685	0.710	39.47	11.85	y	y
Florida State University	44.12	35.71	36.59	20.00	30.00	28.13	33.33	-1.933	1.309	0.200	8.70	8.57	9.09	17.02	13.56	20.00	6.38	0.728	0.998	0.500	33.20	12.59	y	y
Georgia Institute of Technology-Main Campus	46.00	32.81	27.46	36.71	29.70	34.88	32.80	-1.186	1.120	0.340	8.64	10.00	10.84	9.24	14.16	10.46	14.46	0.776	0.328	0.060	33.78	11.17	y	y
Hampton University	100.00	66.67	0.00	100.00	50.00	40.00	42.86	-6.241	6.810	0.400	33.33	x	0.00	41.67	28.57	x	x	0.714	7.470	n/a	53.13	28.85	n	n
Howard University	37.50	60.00	82.35	65.22	56.25	75.00	46.15	1.066	3.200	0.750	35.71	25.00	10.00	7.14	26.67	50.00	10.00	-0.374	3.260	0.910	63.55	24.14	n	y
Iowa State University	28.57	45.74	33.33	33.63	49.51	34.26	30.19	-0.069	1.650	0.970	4.11	6.58	5.97	13.46	12.38	9.38	9.26	0.980	0.555	0.140	36.35	9.23	y	y
Kansas State University	30.77	25.00	22.86	23.81	24.44	35.48	27.08	0.411	0.923	0.67	9.30	8.82	7.89	8.33	5.56	4.76	14.00	0.130	0.618	0.840	26.92	8.42	y	y
Louisiana State University and Agricultural & Mechanical Colleg	27.78	34.85	38.46	27.27	48.00	29.87	38.83	1.170	1.453	0.46	4.29	8.62	5.00	4.84	6.25	16.67	10.71	1.308	0.711	0.130	35.57	7.84	y	y
Louisiana Tech University	20.00	16.67	28.00	21.74	28.00	37.50	14.29	0.876	1.607	0.61	9.38	8.82	22.22	9.38	6.45	25.00	13.33	1.016	1.427	0.510	23.43	12.82	n	n
Massachusetts Institute of Technology	53.95	66.18	56.00	61.19	51.56	44.62	59.70	-1.082	1.375	0.47	40.30	24.56	31.82	25.00	25.00	40.82	26.92	-0.516	1.476	0.740	56.22	30.55	y	y
Michigan Technological University	26.32	24.24	27.40	35.21	32.00	17.95	36.27	0.782	1.299	0.57	8.65	11.76	13.68	8.05	8.49	10.58	12.35	0.125	0.449	0.790	28.93	10.42	y	y
Mississippi State University	28.21	29.27	19.23	14.58	17.95	20.34	30.67	-0.420	1.303	0.76	13.33	7.89	10.71	11.11	12.82	10.26	12.96	0.204	0.388	0.620	23.23	11.36	n	y
Missouri University of Science and Technology	30.00	45.28	35.09	39.66	31.51	28.77	34.95	-0.777	1.154	0.53	7.23	6.33	4.88	13.33	11.49	7.06	10.89	0.681	0.573	0.290	34.59	8.90	y	y
Montana State University	29.63	44.68	47.22	40.38	47.12	31.62	42.86	0.480	1.470	0.76	8.70	16.67	15.79	7.69	7.69	7.32	16.13	-0.161	0.926	0.870	40.58	11.22	n	n
New Mexico Institute of Mining and Technology	18.18	31.25	42.86	31.25	34.78	52.94	30.43	2.574	1.943	0.240	13.64	0.00	8.00	18.18	18.75	17.65	23.08	2.656	1.102	0.061	35.00	13.01	n	n
New Mexico State University-Main Campus	40.00	35.29	52.94	45.00	40.91	48.15	42.11	0.714	1.156	0.56	24.32	20.00	15.15	22.73	16.42	0.00	9.30	-2.993	1.131	0.050	43.71	14.61	n	n
North Carolina A & T State University	57.14	62.50	66.67	33.33	62.50	66.67	45.45	-1.104	2.540	0.68	10.00	30.56	29.73	15.15	20.00	4.17	12.12	-2.005	1.856	0.330	56.73	17.94	n	y
North Carolina State University at Raleigh	32.71	29.27	29.53	25.34	34.18	26.29	36.16	0.322	0.820	0.71	6.87	10.07	8.28	8.00	12.03	9.66	8.61	0.291	0.322	0.400	30.53	9.12	y	y
Northwestern University	43.33	30.00	44.00	25.58	28.21	26.19	38.46	-1.358	1.550	0.420	14.29	18.18	26.32	22.22	16.67	8.33	53.85	3.191	2.715	0.300	32.66	21.54	y	y
Oklahoma State University-Main Campus	36.36	30.00	53.33	30.23	32.56	25.00	27.42	-2.057	1.724	0.290	12.90	6.90	17.24	12.50	4.65	9.52	6.67	-0.930	0.813	0.300	32.55	9.56	y	y
Oregon State University	26.83	38.78	25.00	18.31	25.51	23.86	30.68	-0.634	1.290	0.64	12.12	15.15	3.09	5.13	8.89	15.89	8.70	-0.107	0.999	0.920	26.46	9.69	y	y
Pennsylvania State University-Main Campus	28.04	20.66	25.62	25.17	29.50	25.85	24.38	0.117	0.580	0.85	7.09	4.00	11.25	8.92	11.49	11.24	10.00	0.837	0.432	0.110	25.58	9.30	y	y
Prairie View A & M University	35.29	56.00	54.76	28.57	29.73	36.67	40.32	-1.736	2.170	0.46	27.27	15.38	55.00	8.70	11.11	18.42	21.43	-1.977	3.115	0.550	39.13	21.54	n	y
Princeton University	47.22	51.85	55.88	54.55	54.55	57.14	53.33	0.985	0.510	0.110	29.03	22.73	31.82	36.84	40.00	31.43	48.57	3.007	1.087	0.040	53.64	34.48	y	y
Purdue University-Main Campus	28.87	33.58	31.90	28.99	32.14	31.01	34.25	0.401	0.390	0.350	8.33	10.47	9.58	13.59	12.41	9.90	17.32	1.024	0.438	0.070	31.54	11.60	y	y
Rensselaer Polytechnic Institute	43.14	39.39	23.88	35.62	37.88	28.38	30.49	-1.642	1.198	0.230	11.00	25.97	18.57	8.05	21.43	11.90	23.46	0.432	1.416	0.770	33.61	16.70	y	y
Rose-Hulman Institute of Technology	36.99	26.92	25.49	37.21	33.33	43.06	29.69	0.650	1.270	0.63	7.50	12.50	6.25	6.35	10.20	17.02	15.38	1.309	0.689	0.120	33.73	10.84	n	n
Rowan University	39.13	15.00	27.27	32.35	34.62	26.83	23.08	-0.613	1.630	0.72	10.53	4.76	3.33	16.00	11.11	10.42	5.88	0.184	0.912	0.850	28.29	8.82	n	n
Rutgers University-New Brunswick	41.07	28.81	27.54	23.94	34.29	33.33	34.62	-0.128	1.170	0.92	9.80	7.14	8.78	12.23	7.75	12.50	13.07	0.696	0.393	0.140	32.01	10.37	y	y
San Jose State University	36.84	33.33	27.78	25.00	34.62	20.00	37.74	-0.612	1.350	0.67	8.42	15.28	15.58	12.50	8.45	13.33	5.00	-0.760	0.754	0.360	31.64	10.92	n	n
South Dakota School of Mines and Technology	26.67	28.00	27.78	25.93	37.50	36.11	43.75	2.757	0.713	0.010	0.00	7.69	8.70	11.54	0.00	20.00	15.00	2.176	1.179	0.120	33.51	9.09	y	n
Stanford University	25.00	39.13	52.17	36.36	27.27	35.90	23.33	-1.299	1.997	0.54	19.44	13.95	20.51	25.00	18.18	16.67	10.00	-0.901	0.911	0.370	34.08	17.20	y	y
Texas A & M University-College Station	28.36	31.86	28.95	29.29	31.17	32.08	24.56	-0.312	0.520	0.58	15.91	13.73	13.89	9.85	12.41	14.94	16.33	0.078	0.459	0.870	29.46	13.95	y	y
The Johns Hopkins University	48.15	24.29	39.66	35.38	33.87	31.58	37.04	-0.876	1.500	0.58	0.00	17.65	35.29	8.00	41.18	20.83	26.67	3.295	2.618	0.260	35.84	21.53	y	y
The Ohio State University-Main Campus	23.96	20.87	18.60	28.81	28.41	30.20	26.67	1.307	0.690	0.100	6.04	6.40	11.24	6.43	12.32	14.42	10.73	1.114	0.484	0.070	25.98	10.11	y	y
The University of Alabama	21.43	43.18	31.03	28.57	42.05	32.08	37.21	1.291	1.490	0.420	8.70	15.22	11.32	18.33	13.46	17.86	21.74	1.663	0.552	0.030	34.73	16.05	y	y
The University of Kansas	52.17	30.95	33.93	36.54	35.29	36.36	28.17	-2.137	1.269	0.150	8.33	8.00	21.43	11.76	8.57	11.54	5.00	-0.564	1.062	0.620	35.66	10.27	n	y
The University of Tennessee-Knoxville	25.00	39.29	32.43	26.47	48.98	22.22	22.41	-0.905	2.033	0.67	4.08	2.44	12.50	6.82	7.14	6.82	18.00	1.613	0.831	0.110	30.30	8.53	y	y
The University of Texas at Austin	32.26	36.36	31.25	36.94	43.80	33.92	33.55	0.412	0.850	0.65	14.35	10.61	11.02	12.93	17.47	11.76	12.88	0.155	0.481	0.760	35.46	13.05	y	y
Tuskegee University	92.31	53.85	66.67	42.86	60.00	53.33	73.33	-2.308	3.190	0.5	20.00	33.33	23.53	18.18	23.08	36.84	20.00	0.234	1.489	0.880	62.64	25.86	n	n
University of Arkansas	29.03	27.91	24.14	33.33	28.79	37.50	47.62	2.843	1.017	0.040	16.67	8.33	10.34	9.76	8.33	11.63	8.16	-0.748	0.528	0.220	33.86	10.20	n	n
University of California-Berkeley	38.55	27.91	32.58	31.25	30.65	30.56	22.33	-1.618	0.707	0.070	9.33	8.08	11.25	11.82	10.32	13.90	11.23	0.586	0.283	0.090	30.31	11.01	y	y
University of California-Davis	31.48	29.82	42.86	27.54	35.87	24.21	32.80	-0.509	1.230	0.7	50.00	100.00	0.00	0.00	x	0.00	x	-15.541	7.760	n/a	32.03	13.33	y	y
University of California-Los Angeles	42.86	33.33	30.59	31.76	31.11	25.64	34.15	-1.464	0.860	0.150	17.19	12.14	14.53	15.03	14.57	17.31	16.00	0.243	0.355	0.520	32.84	15.12	y	y
University of California-San Diego	38.46	39.39	19.61	34.21	27.43	35.07	36.27	-0.264	1.450	0.86	16.84	13.13	15.74	15.18	10.53	15.15	8.84	-0.900	0.460	0.110	33.58	13.03	y	y
University of California-Santa Barbara	32.50	30.00	21.28	23.81	36.17	25.49	22.22	-0.891	1.096	0.45	11.11	15.00	5.77	9.76	7.02	8.16	14.06	-0.128	0.719	0.870	26.90	10.06	y	y
University of Colorado Boulder	47.27	29.63	21.15	23.08	39.53	40.00	30.86	-0.361	1.980	0.86	10.34	0.00	15.38	13.16	15.79	14.58	0.00	-0.052	1.448	0.970	33.06	11.49	y	y
University of Connecticut	34.78	36.17	21.74	32.69	16.00	36.59	31.34	-0.544	1.624	0.75	5.71	5.56	5.56	4.55	15.52	18.18	18.03	2.577	0.666	0.011	29.45	11.69	y	y
University of Delaware	26.47	30.61	23.08	30.77	30.26	19.54	18.82	-1.354	0.890	0.190	7.14	6.45	8.00	17.65	20.00	5.56	17.07	1.429	1.120	0.260	24.78	12.50	y	y
University of Florida	29.73	35.37	32.18	29.13	28.28	31.82	35.66	0.243	0.597	0.7	12.64	8.40	10.53	15.85	20.00	16.22	9.09	0.516	0.855	0.570	31.94	13.16	y	y
University of Idaho	35.29	46.15	28.57	10.53	31.03	29.41	36.36	-0.993	2.199	0.67	9.68	14.29	15.38	13.64	12.50	0.00	2.56	-1.886	0.934	0.100	30.53	9.04	y	y
University of Illinois at Urbana-Champaign	32.05	27.27	29.52	34.02	31.67	32.70	27.87	0.016	0.527	0.98	8.91	11.30	14.22	12.86	14.92	11.65	12.00	0.381	0.376	0.360	30.72	12.45	y	y
University of Iowa	20.83	46.15	29.03	24.00	26.92	14.81	27.27	-1.624	1.876	0.43	0.00	10.53	11.11	11.11	14.71	14.94	21.05	2.699	0.545	0.004	28.24	13.65	y	n
University of Kentucky	31.43	31.43	37.14	28.57	31.91	29.49	41.94	0.800	0.914	0.420	12.50	12.00	6.67	8.62	6.35	7.55	13.51	-0.221	0.612	0.730	33.23	9.72	y	y
University of Louisville	37.14	17.39	26.67	29.03	21.43	34.29	30.77	0.337	1.429	0.82	10.42	7.84	2.00	11.11	12.07	16.67	4.35	0.340	1.011	0.750	28.96	9.30	y	y
University of Maine	18.75	34.78	25.00	18.75	23.81	31.03	27.50	0.627	1.207	0.63	0.00	3.70	0.00	8.70	23.08	0.00	4.17	1.006	1.661	0.570	25.93	5.00	y	y
University of Maryland-College Park	36.36	34.48	33.96	37.70	33.33	22.73	34.21	-1.093	0.886	0.270	13.98	22.03	6.92	7.81	14.29	17.50	14.40	-0.016	1.086	0.990	33.02	13.70	y	y
University of Massachusetts-Amherst	33.33	34.15	28.57	18.75	26.09	29.41	17.59	-2.113	0.969	0.080	2.38	8.33	15.22	17.86	11.76	16.00	11.11	1.360	0.908	0.200	25.30	11.73	y	y
University of Michigan-Ann Arbor	30.47	33.10	32.19	32.58	37.78	35.42	32.37	0.569	0.431	0.240	14.13	16.67	18.25	17.89	19.53	21.24	20.19	1.022	0.184	0.003	33.40	18.39	y	y
University of Minnesota-Duluth	26.92	15.38	27.50	11.54	15.79	18.18	34.09	0.550	1.681	0.76	10.00	0.00	4.76	11.90	0.00	6.06	13.64	0.652	1.096	0.580	21.96	7.63	n	n
University of Minnesota-Twin Cities	35.00	21.43	19.83	22.64	25.00	22.11	28.07	-0.510	1.050	0.65	5.69	6.56	16.80	8.57	15.04	12.32	8.73	0.674	0.830	0.450	24.80	10.48	y	y
University of Mississippi	33.33	50.00	35.29	43.75	57.89	28.95	29.03	-1.157	2.245	0.63	21.43	14.29	28.57	16.67	26.67	10.00	0.00	-2.670	1.675	0.170	37.50	16.46	n	n
University of Nebraska-Lincoln	35.29	5.88	21.43	20.69	27.59	20.69	21.88	-0.160	1.834	0.93	12.00	3.39	4.35	8.77	16.42	8.77	4.48	0.010	0.980	0.990	22.10	8.44	y	y
University of Nevada-Reno	30.00	21.43	20.00	40.00	31.82	21.74	22.73	-0.335	1.521	0.83	17.39	16.00	22.73	16.00	3.45	17.78	11.11	-1.234	1.137	0.330	26.98	14.63	y	y
University of New Hampshire-Main Campus	27.78	27.27	21.88	25.81	31.25	22.22	31.25	0.346	0.773	0.67	5.00	5.71	16.67	12.12	6.90	6.06	8.00	-0.003	0.880	0.990	26.96	8.54	y	y
University of North Dakota	16.13	10.71	28.57	13.04	20.00	16.67	29.73	1.576	1.351	0.300	12.00	7.14	6.25	0.00	2.70	13.64	14.04	0.555	1.107	0.640	19.10	8.49	y	n
University of Oklahoma-Norman Campus	38.10	33.33	38.18	36.99	30.00	35.29	39.13	-0.041	0.673	0.95	3.85	11.54	16.13	9.09	14.63	11.48	15.09	1.147	0.714	0.170	36.17	12.06	y	y
University of Pennsylvania	34.69	44.12	33.33	34.78	54.29	47.06	26.19	0.047	1.990	0.98	3.23	20.83	19.23	10.34	32.00	27.78	25.00	3.285	1.471	0.076	38.25	18.34	y	y
University of Pittsburgh-Pittsburgh Campus	28.00	24.07	45.33	27.03	23.23	28.87	36.11	0.422	1.610	0.8	10.00	14.55	5.26	13.46	12.73	13.21	15.79	0.791	0.636	0.270	30.70	12.14	y	y
University of Puerto Rico-Mayaguez	71.43	62.22	59.13	58.62	50.57	53.62	53.49	-2.842	0.692	0.009	19.82	21.95	18.82	16.13	12.00	25.64	19.75	0.013	0.891	0.990	58.58	19.17	y	n
University of Rhode Island	30.00	41.94	43.48	33.33	25.93	24.00	31.25	-1.774	1.320	0.240	14.29	11.11	8.70	14.81	0.00	9.09	2.86	-1.679	0.868	0.110	32.98	9.00	y	y
University of South Carolina-Columbia	25.93	29.03	20.59	30.77	33.33	38.46	33.33	1.922	0.831	0.070	12.50	0.00	16.67	6.90	0.00	18.37	11.11	0.568	1.511	0.720	30.71	9.88	y	y
University of Utah	30.77	16.67	13.33	12.96	21.05	19.05	25.45	-0.124	1.340	0.93	19.51	2.17	3.77	6.06	8.51	5.26	13.46	-0.258	1.260	0.850	19.68	8.01	y	y
University of Washington-Seattle Campus	43.33	37.04	31.75	28.57	36.62	35.00	26.67	-1.757	0.870	0.100	11.59	17.31	14.29	11.49	24.16	23.16	21.74	1.858	0.751	0.060	33.91	18.04	y	y
University of Wisconsin-Madison	30.61	26.76	16.47	28.42	21.35	26.32	32.29	0.322	1.120	0.78	10.00	13.85	3.33	6.35	14.52	12.66	19.12	1.291	0.936	0.230	25.85	11.60	y	y
University of Wyoming	23.81	25.00	25.00	18.92	13.79	35.71	23.53	0.335	1.377	0.82	6.25	4.35	11.54	5.56	9.52	0.00	6.25	-0.383	0.745	0.630	23.28	6.77	y	y
Vanderbilt University	44.83	50.00	51.02	48.94	40.35	50.88	34.69	-1.404	1.132	0.270	35.71	20.00	31.82	25.00	26.92	21.43	22.22	-1.518	0.988	0.180	45.78	26.35	y	y
Virginia Polytechnic Institute and State University	29.82	24.76	41.77	34.95	17.71	33.64	41.30	1.005	1.750	0.59	8.74	14.00	11.11	8.41	13.19	8.59	11.67	0.002	0.475	0.990	31.77	10.86	y	y
Washington State University	23.53	19.35	36.84	22.86	33.33	30.77	17.86	0.082	1.510	0.96	13.33	5.88	5.97	11.69	2.17	11.21	11.46	0.044	0.854	0.960	25.42	8.91	y	y
Washington University in St Louis	41.38	25.81	21.88	32.35	30.56	43.33	32.20	0.579	1.580	0.73	11.11	0.00	22.22	28.57	32.14	17.95	10.53	1.574	2.213	0.510	33.45	19.19	n	y
West Virginia University	40.00	26.67	30.30	33.33	29.73	27.91	32.20	-0.767	0.847	0.400	9.76	20.00	7.50	14.29	6.98	13.95	12.00	-0.210	0.935	0.830	30.71	11.40	n	n
West Virginia University Institute of Technology	0.00	42.86	45.45	0.00	20.00	44.44	25.00	1.883	4.040	0.66	14.29	0.00	22.22	0.00	0.00	20.00	16.67	0.890	2.050	0.680	35.90	10.29	n	n
Worcester Polytechnic Institute	36.84	41.30	39.73	40.58	52.31	45.19	42.86	1.372	0.826	0.160	10.26	9.46	17.58	13.95	21.43	17.53	21.36	1.903	0.545	0.017	42.97	16.43	y	y
Yale University	40.00	100.00	36.36	37.50	28.57	30.00	50.00	-4.207	4.780	0.420	0.00	0.00	0.00	33.33	50.00	60.00	0.00	6.071	4.814	0.260	39.29	17.86	y	y
sum of focal	35.06	33.15	32.19	32.17	33.41	32.10	33.19	-0.232	0.170	0.280	11.43	11.63	12.20	11.67	13.11	13.62	13.73	0.422	0.098					
ASEE	34.50	33.10	32.00	32.30	36.30	32.40	33.30	-0.025	0.315		11.60	11.50	12.70	12.30	13.70	12.50	12.70	0.225	0.117					
NSF	32.11	30.62	30.38	29.80	30.67	29.40		-0.400	0.150		10.68	10.56	11.06	11.23	11.66	11.90		0.273	0.036					

S1 Table: Institution Data

	ABET 2005				ABET 2010				ABET 2015													
Institution Name	# total TT faculty, ChE	# female TT faculty, ChE	# total TT faculty, EE	# female TT faculty, EE	# total TT faculty, ChE	# female TT faculty, ChE	# total TT faculty, EE	# female TT faculty, EE	# total TT faculty, ChE	# female TT faculty, ChE	# total TT faculty, EE	# female TT faculty, EE	ave # TT, ChE	ave TT % female, ChE	ave # TT, EE	ave TT% female, EE	state	Census Region	public or private?	**HBCU?**	**ChE dept name**	**EE dept name**
Arizona State University-Tempe	22	1	54	5	13	5	56	9	17	6	69	14	17.33	26.10	59.67	15.21	AZ	West: Mountain	public	non-HBCU	School for Engineering of Matter, Transport & Energy	School of Electrical, Computer and Energy Engineering
Brigham Young University-Provo	12	0	25	0	14	0	23	0	17	0	23	0	14.33	0.00	23.67	0.00	UT	West: Mountain	private	non-HBCU	Chemical Engineering	Electrical and Computer Engineering
California Institute of Technology	11	3	14	2	11	3	12	3	11	2	12	2	11.00	24.24	12.67	18.65	CA	West: Pacific	private	non-HBCU	Chemistry and Chemical Engineering	Electrical Engineering
California State Polytechnic University-Pomona	6	1	28	5	5	1	20	5	7	2	20	6	6.00	21.75	22.67	24.29	CA	West: Pacific	public	non-HBCU	Chemical & Materials Engineering	Electrical & Computer Engineering
California State University-Long Beach	4	1	18	1	4	1	19	2	6	1	19	3	4.67	22.22	18.67	10.62	CA	West: Pacific	public	non-HBCU	Chemical Engineering	Electrical Engineering
Carnegie Mellon University	15	0	41	4	19	3	46	3	19	4	49	4	17.67	12.28	45.33	8.15	PA	Northeast: Mid-Atlantic	private	non-HBCU	Chemical Engineering	Electrical and Computer Engineering
Case Western Reserve University	14	1	35	2	11	1	33	2	10	1	32	3	11.67	8.74	33.33	7.05	OH	Midwest: East North Central	private	non-HBCU	Chemical and Biomolecular Engineering	Electrical Engineering
Clemson University	13	0	29	1	10	0	31	3	12	2	26	2	11.67	5.56	28.67	6.94	SC	South: South Atlantic	public	non-HBCU	Chemical & Biomolecular Engineering	Electrical and Computer Engineering
Colorado State University-Fort Collins	7	0	18	1	11	1	25	2	12	1	27	4	10.00	5.81	23.33	9.46	CO	West: Mountain	public	non-HBCU	Chemical and Biological Engineering	Electrical and Computer Engineering
Cooper Union for the Advancement of Science and Art	5	1	6	0	5	0	7	0	5	0	5	0	5.00	6.67	6.00	0.00	NY	Northeast: Mid-Atlantic	private	non-HBCU	Chemical Engineering	Electrical Engineering
Cornell University	15	1	39	5	16	2	38	3	20	3	33	2	17.00	11.39	36.67	8.93	NY	Northeast: Mid-Atlantic	private	non-HBCU	Chemical and Biomolecular Engineering	Electrical and Computer Engineering
Drexel University	10	0	34	4	13	2	32	4	14	3	32	4	12.33	12.27	32.67	12.25	PA	Northeast: Mid-Atlantic	private	non-HBCU	Chemical & Biological Engineering	Electrical and Computer Engineering
Florida State University	n/a	n/a	n/a	n/a	n/a	n/a	n/a	n/a	n/a	n/a	n/a	n/a	n/a	n/a	n/a	n/a	FL	South: South Atlantic	public	non-HBCU	Chemical & Biomedical Engineering	Electrical & Computer Engineering
Georgia Institute of Technology-Main Campus	30	3	101	9	36	9	108	12	34	8	111	15	33.33	19.51	106.67	11.18	GA	South: South Atlantic	public	non-HBCU	Chemical & Biomolecular Engineering	Electrical and Computer Engineering
Hampton University	n/a	n/a	n/a	n/a	n/a	n/a	n/a	n/a	n/a	n/a	n/a	n/a	n/a	n/a	n/a	n/a	VA	South: South Atlantic	private	HBCU	Chemical Engineering	Electrical Engineering
Howard University	4	0	12	0	6	0	10	0	5	1	11	0	5.00	6.67	11.00	0.00	DC	South: South Atlantic	private	HBCU	Chemical Engineering	Electrical and Computer Engineering
Iowa State University	16	3	48	4	18	5	53	5	20	5	59	8	18.00	23.84	53.33	10.44	IA	Midwest: West North Central	public	non-HBCU	Chemical and Biological Engineering	Electrical and Computer Engineering
Kansas State University	10	2	21	3	11	2	18	4	10	2	20	3	10.33	19.39	19.67	17.17	KS	Midwest: West North Central	public	non-HBCU	Chemical Engineering	Electrical & Computer Engineering
Louisiana State University and Agricultural & Mechanical Colleg	13	2	21	0	16	1	26	1	15	0	24	0	14.67	7.21	23.67	1.28	LA	South: West South Central	public	non-HBCU	Chemical Engineering	Electrical & Computer Engineering
Louisiana Tech University	4	2	11	1	4	2	8	1	5	1	4	0	4.33	40.00	7.67	7.20	LA	South: West South Central	public	non-HBCU	Chemical Engineering	Electrical Engineering
Massachusetts Institute of Technology	30	4	127	16	31	4	126	17	34	5	122	20	31.67	13.65	125.00	14.16	MA	Northeast: New England	private	non-HBCU	Chemical Engineering	Electrical Engineering and Computer Science
Michigan Technological University	14	2	19	3	15	4	20	4	11	3	24	4	13.33	22.74	21.00	17.49	MI	Midwest: East North Central	public	non-HBCU	Chemical Engineering	Electrical and Computer Engineering
Mississippi State University	11	4	25	3	8	3	20	1	9	2	18	1	9.33	32.03	21.00	7.52	MS	South: East South Central	public	non-HBCU	Chemical Engineering	Electrical and Computer Engineering
Missouri University of Science and Technology	11	2	30	3	9	0	30	4	13	2	30	5	11.00	11.19	30.00	13.33	MO	Midwest: West North Central	public	non-HBCU	Chemical and Biochemical Engineering	Electrical and Computer Engineering
Montana State University	8	0	14	0	10	2	13	0	14	6	13	1	10.67	20.95	13.33	2.56	MT	West: Mountain	public	non-HBCU	Chemical & Biological Engineering	Electrical & Computer Engineering
New Mexico Institute of Mining and Technology	n/a	n/a	n/a	n/a	3	1	8	0	5	2	7	0	4.00	36.67	7.50	0.00	NM	West: Mountain	public	non-HBCU	Chemical Engineering	Electrical Engineering
New Mexico State University-Main Campus	7	0	21	1	10	3	19	1	8	4	17	1	8.33	26.67	19.00	5.30	NM	West: Mountain	public	non-HBCU	Chemical & Materials Engineering	Electrical and Computer Engineering
North Carolina A & T State University	8	0	17	1	4	n/a	16	1	10	0	15	1	7.33	0.00	16.00	6.27	NC	South: South Atlantic	public	HBCU	Chemical, Biological and Bio Engineering	Electrical and Computer Engineering
North Carolina State University at Raleigh	21	2	51	4	16	2	50	4	25	2	55	5	20.67	10.01	52.00	8.31	NC	South: South Atlantic	public	non-HBCU	Chemical and Biomolecular Engineering	Electrical & Computer Engineering
Northwestern University	13	2	44	3	12	1	50	3	15	3	53	5	13.33	14.57	49.00	7.42	IL	Midwest: East North Central	private	non-HBCU	Chemical & Biological Engineering	Electrical Engineering & Computer Science
Oklahoma State University-Main Campus	11	1	23	0	13	2	26	2	13	2	23	1	12.33	13.29	24.00	4.01	OK	South: West South Central	public	non-HBCU	Chemical Engineering	Electrical and Computer Engineering
Oregon State University	12	2	39	6	8	1	21	3	24	4	52	8	14.67	15.28	37.33	15.02	OR	West: Pacific	public	non-HBCU	Chemical, Biological, and Environmental Engineering	Electrical and Computer Engineering
Pennsylvania State University-Main Campus	18	3	37	5	17	2	35	3	20	3	58	5	18.33	14.48	43.33	10.24	PA	Northeast: Mid-Atlantic	public	non-HBCU	Chemical Engineering	Electrical Engineering and Computer Science
Prairie View A & M University	6	1	13	1	4	1	12	2	1	0	16	4	3.67	13.89	13.67	16.45	TX	South: West South Central	public	HBCU	Chemical Engineering	Electrical and Computer Engineering
Princeton University	18	2	31	4	17	2	28	3	16	2	29	2	17.00	11.79	29.33	10.17	NJ	Northeast: Mid-Atlantic	private	non-HBCU	Chemical and Biological Engineering	Electrical Engineering
Purdue University-Main Campus	24	2	81	8	22	4	77	8	23	3	77	12	23.00	13.19	78.33	11.95	IN	Midwest: East North Central	public	non-HBCU	Chemical Engineering	Electrical and Computer Engineering
Rensselaer Polytechnic Institute	12	1	41	5	13	1	38	5	11	1	33	7	12.00	8.37	37.33	15.52	NY	Northeast: Mid-Atlantic	private	non-HBCU	Chemical & Biological Engineering	Electrical, Computer, and Systems Engineering
Rose-Hulman Institute of Technology	8	1	20	2	9	2	20	5	10	3	16	3	9.00	21.57	18.67	17.92	IN	Midwest: East North Central	private	non-HBCU	Chemical Engineering	Electrical & Computer Engineering
Rowan University	8	2	8	2	9	5	8	2	8	4	7	3	8.33	43.52	7.67	30.95	NJ	Northeast: Mid-Atlantic	public	non-HBCU	Chemical Engineering	Electrical and Computer Engineering
Rutgers University-New Brunswick	14	1	28	3	14	3	29	5	19	4	34	8	15.67	16.54	30.33	17.16	NJ	Northeast: Mid-Atlantic	public	non-HBCU	Chemical & Biochemical Engineering	Electrical & Computer Engineering
San Jose State University	7	1	23	1	7	3	16	2	2	0	3	1	5.33	19.05	14.00	16.73	CA	West: Pacific	public	non-HBCU	Biomedical, Chemical & Materials Engineering	Electrical Engineering
South Dakota School of Mines and Technology	9	1	8	1	12	1	6	0	9	1	6	1	10.00	10.19	6.67	9.72	SD	Midwest: West North Central	public	non-HBCU	Chemical and Biological Engineering	Electrical and Computer Engineering
Stanford University	10	3	49	3	13	2	48	5	14	3	50	5	12.33	22.27	49.00	8.85	CA	West: Pacific	private	non-HBCU	Chemical Engineering	Electrical Engineering
Texas A & M University-College Station	23	2	56	6	24	5	59	6	30	3	71	7	25.67	13.18	62.00	10.25	TX	South: West South Central	public	non-HBCU	Chemical Engineering	Electrical & Computer Engineering
The Johns Hopkins University	11	1	19	0	13	2	17	2	14	3	20	4	12.67	15.30	18.67	10.59	MD	South: South Atlantic	private	non-HBCU	Chemical & Biomolecular Engineering	Electrical and Computer Engineering
The Ohio State University-Main Campus	15	2	46	4	20	3	48	3	20	4	43	5	18.33	16.11	45.67	8.86	OH	Midwest: East North Central	public	non-HBCU	Chemical and Biomolecular Engineering	Electrical and Computer Engineering
The University of Alabama	11	1	11	0	12	2	17	4	13	3	19	3	12.00	16.28	15.67	13.11	AL	South: East South Central	public	non-HBCU	Chemical and Biological Engineering	Electrical and Computer Engineering
The University of Kansas	18	4	35	4	17	4	31	3	19	4	30	4	18.00	22.27	32.00	11.48	KS	Midwest: West North Central	public	non-HBCU	Chemical and Petroleum Engineering	Electrical Engineering and Computer Science
The University of Tennessee-Knoxville	10	1	24	2	11	1	35	4	15	0	41	7	12.00	6.36	33.33	12.28	TN	South: East South Central	public	non-HBCU	Chemical & Biomolecular Engineering	Electrical Engineering & Computer Science
The University of Texas at Austin	21	1	59	4	21	1	64	4	25	4	60	4	22.33	8.51	61.00	6.57	TX	South: West South Central	public	non-HBCU	Chemical Engineering	Electrical and Computer Engineering
Tuskegee University	4	1	3	0	6	0	14	4	6	3	9	1	5.33	25.00	8.67	13.23	AL	South: East South Central	private	HBCU	Chemical Engineering	Electrical Engineering
University of Arkansas	10	0	22	2	14	2	17	2	12	2	15	2	12.00	10.32	18.00	11.40	AR	South: West South Central	public	non-HBCU	Chemical Engineering	Electrical Engineering
University of California-Berkeley	16	3	84	5	19	5	82	7	17	4	93	12	17.33	22.87	86.33	9.13	CA	West: Pacific	public	non-HBCU	Chemical & Biomolecular Engineering	Electrical Engineering and Computer Sciences
University of California-Davis	31	8	33	3	31	9	29	5	31	9	30	5	31.00	27.96	30.67	14.33	CA	West: Pacific	public	non-HBCU	Chemical Engineering	Electrical and Computer Engineering
University of California-Los Angeles	12	2	45	2	12	2	45	5	13	3	46	7	12.33	18.80	45.33	10.26	CA	West: Pacific	public	non-HBCU	Chemical and Biomolecular Engineering	Electrical and Computer Engineering
University of California-San Diego	n/a	n/a	53	2	14	4	49	2	23	4	51	6	18.50	22.98	51.00	6.54	CA	West: Pacific	public	non-HBCU	NanoEngineering	Electrical and Computer Engineering
University of California-Santa Barbara	20	1	38	3	17	1	37	3	17	4	41	3	18.00	11.47	38.67	7.77	CA	West: Pacific	public	non-HBCU	Chemical Engineering	Electrical and Computer Engineering
University of Colorado Boulder	18	4	38	4	23	5	39	7	23	6	36	4	21.33	23.35	37.67	13.20	CO	West: Mountain	public	non-HBCU	Chemical and Biological Engineering	Electrical, Computer & Energy Engineering
University of Connecticut	11	0	12	0	17	1	17	3	18	5	22	2	15.33	11.22	17.00	8.91	CT	Northeast: New England	public	non-HBCU	Chemical & Biomolecular Engineering	Electrical and Computer Engineering
University of Delaware	20	2	23	1	25	4	25	3	22	3	20	2	22.33	13.21	22.67	8.78	DE	South: South Atlantic	public	non-HBCU	Chemical and Biomolecular Engineering	Electrical & Computer Engineering
University of Florida	20	1	45	3	21	4	40	5	21	1	51	5	20.67	9.60	45.33	9.66	FL	South: South Atlantic	public	non-HBCU	Chemical Engineering	Electrical and Computer Engineering
University of Idaho	8	0	17	0	9	0	15	1	13	2	8	0	10.00	5.13	13.33	2.22	ID	West: Mountain	public	non-HBCU	Chemical & Materials Engineering	Electrical & Computer Engineering
University of Illinois at Urbana-Champaign	13	1	88	5	14	2	78	5	18	3	79	9	15.00	12.88	81.67	7.83	IL	Midwest: East North Central	public	non-HBCU	Chemical and Biomolecular Engineering	Electrical and Computer Engineering
University of Iowa	11	2	14	0	11	3	17	1	11	3	21	4	11.00	24.24	17.33	8.31	IA	Midwest: West North Central	public	non-HBCU	Chemical and Biochemical Engineering	Electrical and Computer Engineering
University of Kentucky	21	4	23	2	23	2	23	2	25	5	22	1	23.00	15.91	22.67	7.31	KY	South: East South Central	public	non-HBCU	Chemical and Materials Engineering	Electrical & Computer Engineering
University of Louisville	8	2	15	0	8	2	15	2	9	2	14	2	8.33	24.07	14.67	9.21	KY	South: East South Central	public	non-HBCU	Chemical Engineering	Electrical & Computer Engineering
University of Maine	14	0	11	1	12	0	12	1	11	0	13	1	12.33	0.00	12.00	8.37	ME	Northeast: New England	public	non-HBCU	Chemical and Biomedical Engineering	Electrical & Computer Engineering
University of Maryland-College Park	14	2	63	5	12	1	57	3	14	3	59	5	13.33	14.68	59.67	7.22	MD	South: South Atlantic	public	non-HBCU	Chemical and Biomolecular Engineering	Electrical and Computer Engineering
University of Massachusetts-Amherst	12	2	33	2	16	2	31	2	18	5	35	2	15.33	18.98	33.00	6.08	MA	Northeast: New England	public	non-HBCU	Chemical Engineering	Electrical and Computer Engineering
University of Michigan-Ann Arbor	18	2	88	7	20	5	49	5	22	6	64	9	20.00	21.13	67.00	10.74	MI	Midwest: East North Central	public	non-HBCU	Chemical Engineering	Electrical Engineering and Computer Science
University of Minnesota-Duluth	6	0	9	1	6	0	10	2	8	1	11	2	6.67	4.17	10.00	16.43	MN	Midwest: West North Central	public	non-HBCU	Chemical Engineering	Electrical Engineering
University of Minnesota-Twin Cities	32	4	42	2	16	1	42	1	35	5	45	4	27.67	11.01	43.00	5.34	MN	Midwest: West North Central	public	non-HBCU	Chemical Engineering and Materials Science	Electrical and Computer Engineering
University of Mississippi	6	0	11	0	6	0	12	0	8	0	10	0	6.67	0.00	11.00	0.00	MS	South: East South Central	public	non-HBCU	Chemical Engineering	Electrical Engineering
University of Nebraska-Lincoln	12	2	18	0	13	2	18	3	13	2	35	3	12.67	15.81	23.67	8.41	NE	Midwest: West North Central	public	non-HBCU	Chemical & Biomolecular Engineering	Electrical & Computer Engineering
University of Nevada-Reno	11	2	10	2	10	1	11	0	11	0	12	1	10.67	9.39	11.00	9.44	NV	West: Mountain	public	non-HBCU	Chemical and Materials Engineering	Electrical & Biomedical Engineering
University of New Hampshire-Main Campus	7	1	13	1	7	2	10	0	8	2	11	1	7.33	22.62	11.33	5.59	NH	Northeast: New England	public	non-HBCU	Chemical Engineering	Electrical and Computer Engineering
University of North Dakota	3	0	6	0	9	0	7	1	7	1	6	2	6.33	4.76	6.33	15.87	ND	Midwest: West North Central	public	non-HBCU	Chemical Engineering	Electrical Engineering
University of Oklahoma-Norman Campus	15	0	23	1	16	1	25	0	14	0	28	1	15.00	2.08	25.33	2.64	OK	South: West South Central	public	non-HBCU	Chemical, Biological and Materials Engineering	Electrical and Computer Engineering
University of Pennsylvania	11	0	19	1	13	1	23	2	14	2	24	2	12.67	7.33	22.00	7.43	PA	Northeast: Mid-Atlantic	public	non-HBCU	Chemical and Biomolecular Engineering	Electrical and Systems Engineering
University of Pittsburgh-Pittsburgh Campus	14	2	21	0	18	3	17	1	22	4	21	3	18.00	16.38	19.67	6.72	PA	Northeast: Mid-Atlantic	public	non-HBCU	Chemical and Petroleum Engineering	Electrical and Computer Engineering
University of Puerto Rico-Mayaguez	19	1	34	3	28	5	34	2	22	7	41	5	23.00	18.31	36.33	8.97	PR	n/a	public	non-HBCU	Chemical Engineering	Electrical and Computer Engineering
University of Rhode Island	9	1	19	2	11	1	17	3	8	2	20	2	9.33	15.07	18.67	12.72	RI	Northeast: New England	public	non-HBCU	Chemical Engineering	Electrical, Computer and Biomedical Engineering
University of South Carolina-Columbia	18	1	16	1	20	2	15	0	20	2	18	0	19.33	8.52	16.33	2.08	SC	South: South Atlantic	public	non-HBCU	Chemical Engineering	Electrical Engineering
University of Utah	15	2	18	2	14	1	32	4	18	3	31	4	15.67	12.38	27.00	12.17	UT	West: Mountain	public	non-HBCU	Chemical Engineering	Electrical & Computer Engineering
University of Washington-Seattle Campus	17	1	38	5	18	0	41	7	18	4	38	5	17.67	9.37	39.00	14.46	WA	West: Pacific	public	non-HBCU	Chemical Engineering	Electrical Engineering
University of Wisconsin-Madison	18	1	43	5	19	1	38	5	19	2	37	7	18.67	7.12	39.33	14.57	WI	Midwest: East North Central	public	non-HBCU	Chemical & Biological Engineering	Electrical & Computer Engineering
University of Wyoming	18	2	10	1	15	1	12	2	18	2	10	1	17.00	9.63	10.67	12.22	WY	West: Mountain	public	non-HBCU	Chemical Engineering	Electrical and Computer Engineering
Vanderbilt University	10	2	36	3	11	2	33	4	14	3	31	4	11.67	19.87	33.33	11.12	TN	South: East South Central	private	non-HBCU	Chemical and Biomolecular Engineering	Electrical Engineering and Computer Science
Virginia Polytechnic Institute and State University	11	2	61	4	13	2	64	3	15	0	64	5	13.00	11.19	63.00	6.35	VA	South: South Atlantic	public	non-HBCU	Chemical Engineering	Electrical and Computer Engineering
Washington State University	10	2	40	4	16	3	29	2	17	3	41	4	14.33	18.80	36.67	8.88	WA	West: Pacific	public	non-HBCU	Chemical Engineering and Bioengineering	Electrical Engineering & Computer Science
Washington University in St Louis	8	0	15	0	16	2	13	1	18	2	14	2	14.00	7.87	14.00	7.33	MO	Midwest: West North Central	private	non-HBCU	Energy, Environmental & Chemical Engineering	Electrical & Systems Engineering
West Virginia University	10	0	31	5	13	2	33	5	12	1	36	8	11.67	7.91	33.33	17.83	WV	South: South Atlantic	public	non-HBCU	Chemical and Biomedical Engineering	Computer Science and Electrical Engineering
West Virginia University Institute of Technology	4	0	6	0	3	1	6	0	4	2	5	0	3.67	27.78	5.67	0.00	WV	South: South Atlantic	public	non-HBCU	Chemical Engineering	Electrical Engineering
Worcester Polytechnic Institute	10	3	20	2	10	2	17	1	13	4	18	0	11.00	26.92	18.33	5.29	MA	Northeast: New England	private	non-HBCU	Chemical Engineering	Electrical and Computer Engineering
Yale University	9	1	14	1	14	3	15	0	14	4	15	1	12.33	20.37	14.67	4.60	CT	Northeast: New England	private	non-HBCU	Chemical and Environmental Engineering	Electrical Engineering
sum of focal																						
ASEE																						
NSF																						

S1 Table: Institution Data

ABET 2017

	total other programs	Aeronautical Eng Tech	Aerospace Eng	Agricultural Eng	Architectural Eng	Bioeng, Biomed Eng	Biological Eng	Ceramic Eng	Civil Eng (Tech)	Comp Eng (Tech)	Comp Sci	Const Eng (Tech)	Eng Mgmt	Eng Mech	Eng Tech, Eng, Eng Phys, Eng Sci Eng	Envir Eng	Fire Pro Eng (Tech)	Geol Eng	Health Physics	Indust Eng (Tech, Hygeine)	Inform Sys Tech	Manuf Eng (Tech)	Mat Eng	Mech Eng (Tech)	Metal	Mining	Naval Arch and Marine	Nucl and Radiol	Ocean	Optics	Petrol eum	Softw are	Surveying, Geomatics (Eng, Tech)	Systems	Welding
Arizona State University-Tempe	14		x			x			x	x	x	x	x		x					x	x	x	x	x								x			
Brigham Young University-Provo	6								x	x	x										x	x		x											
California Institute of Technology	1																							x											
California State Polytechnic University-Pomon	10		x						x	x	x	x			x					x		x		x									x		
California State University-Long Beach	5		x						x	x	x													x											
Carnegie Mellon University	5								x	x					x								x	x											
Case Western Reserve University	9		x			x			x	x	x				x								x	x										x	
Clemson University	9					x	x		x	x	x					x				x			x	x											
Colorado State University-Fort Collins	7					x	x		x	x					x	x								x											
Cooper Union for the Advancement of Science	2								x															x											
Cornell University	6						x		x	x						x							x	x											
Drexel University	10					x			x	x	x				x	x					x		x	x								x			
Florida State University	5								x	x	x									x				x											
Georgia Institute of Technology-Main Campus	10		x			x			x	x	x					x				x			x	x				x							
Hampton University	1										x																								
Howard University	4								x	x	x													x											
Iowa State University	10		x				x		x	x	x	x								x			x	x								x			
Kansas State University	6						x		x	x	x									x				x											
Louisiana State University and Agricultural & M	8						x		x	x	x					x				x				x							x				
Louisiana Tech University	6					x			x		x	x								x				x											
Massachusetts Institute of Technology	11		x				x		x	x	x				x	x							x	x				x	x						
Michigan Technological University	9					x			x	x					x	x		x					x	x									x		
Mississippi State University	8		x				x		x	x	x									x				x								x			
Missouri University of Science and Technology	13		x					x	x	x	x		x			x		x						x	x	x		x			x				
Montana State University	8						x		x	x	x	x	x							x				x											
New Mexico Institute of Mining and Technolo	7								x		x					x							x	x		x					x				
New Mexico State University-Main Campus	9		x						x	x	x				x					x	x			x									x		
North Carolina A & T State University	9					x	x		x	x	x									x				x									x	x	
North Carolina State University at Raleigh	12		x			x	x		x	x	x	x				x				x			x	x				x							
Northwestern University	8					x			x	x						x				x		x	x	x											
Oklahoma State University-Main Campus	8		x				x		x	x		x					x			x				x											
Oregon State University	11			x		x			x	x	x					x			x	x		x		x				x							
Pennsylvania State University-Main Campus	14		x		x	x	x		x	x					x	x				x			x	x		x		x			x				
Prairie View A & M University	4								x	x	x													x											
Princeton University	3		x						x															x											
Purdue University-Main Campus	16	x	x	x		x	x		x	x		x			x	x				x	x	x	x	x				x							
Rensselaer Polytechnic Institute	10		x			x			x	x			x			x				x			x	x				x							
Rose-Hulman Institute of Technology	8					x			x	x	x				x									x						x		x			
Rowan University	5								x	x	x										x			x											
Rutgers University-New Brunswick	7					x			x	x						x				x			x	x											
San Jose State University	8		x			x			x	x	x									x			x	x											
South Dakota School of Mines and Technology	9								x	x	x		x					x		x				x	x	x									
Stanford University	2								x															x											
Texas A & M University-College Station	12		x	x			x		x	x	x									x		x		x				x	x		x				
The Johns Hopkins University	8					x			x	x	x			x		x							x	x											
The Ohio State University-Main Campus	12		x	x		x	x		x	x	x					x				x			x	x											x
The University of Alabama	9		x						x	x	x	x								x				x	x					x					
The University of Kansas	9		x		x				x	x	x				x						x			x							x				
The University of Tennessee-Knoxville	10		x			x	x		x	x	x									x			x	x				x							
The University of Texas at Austin	8		x		x	x			x	x								x						x							x				
Tuskegee University	3		x								x													x											
University of Arkansas	7					x	x		x	x	x									x				x											
University of California-Berkeley	7								x	x	x									x			x	x				x							
University of California-Davis	8		x			x	x		x	x	x												x	x											
University of California-Los Angeles	7		x			x			x	x	x												x	x											
University of California-San Diego	3		x			x																		x											
University of California-Santa Barbara	3									x	x													x											
University of Colorado Boulder	8		x		x		x		x	x	x					x								x											
University of Connecticut	9					x			x	x	x		x			x						x	x	x											
University of Delaware	5					x			x	x						x								x											
University of Florida	12		x	x			x		x	x						x				x			x	x				x					x	x	
University of Idaho	7			x			x		x	x	x												x	x											
University of Illinois at Urbana-Champaign	13		x	x		x	x		x	x	x			x	x					x			x	x				x							
University of Iowa	4					x			x											x				x											
University of Kentucky	7						x		x	x	x												x	x		x									
University of Louisville	6					x			x	x	x									x				x											
University of Maine	8					x			x	x	x	x			x									x									x		
University of Maryland-College Park	7		x			x			x	x							x						x	x											
University of Massachusetts-Amherst	4								x	x										x				x											
University of Michigan-Ann Arbor	11		x			x			x	x	x					x				x			x	x			x	x							
University of Minnesota-Duluth	5								x	x	x									x				x											
University of Minnesota-Twin Cities	10		x			x	x		x	x							x		x				x	x										x	
University of Mississippi	4								x		x							x						x											
University of Nebraska-Lincoln	7			x			x		x	x	x	x												x											
University of Nevada-Reno	8								x	x	x					x		x						x	x	x									
University of New Hampshire-Main Campus	6								x	x	x					x					x			x											
University of North Dakota	5								x		x							x						x							x				
University of Oklahoma-Norman Campus	10		x		x				x	x	x				x	x				x				x							x				
University of Pennsylvania	6					x				x	x												x	x										x	
University of Pittsburgh-Pittsburgh Campus	7					x			x	x					x					x			x	x											
University of Puerto Rico-Mayaguez	4								x	x										x				x											
University of Rhode Island	6					x			x	x										x				x					x						
University of South Carolina-Columbia	6					x			x	x	x										x			x											
University of Utah	8					x			x	x								x					x	x	x	x									
University of Washington-Seattle Campus	7		x			x			x	x										x			x	x											
University of Wisconsin-Madison	9					x	x		x	x				x				x					x	x				x							
University of Wyoming	6				x				x	x	x													x							x				
Vanderbilt University	5					x			x	x	x													x											
Virginia Polytechnic Institute and State Univer	12		x				x		x	x	x	x		x						x			x	x		x			x						
Washington State University	6					x			x	x	x												x	x											
Washington University in St Louis	3					x																		x										x	
West Virginia University	7								x	x	x									x				x		x					x				
West Virginia University Institute of Technolog	4								x	x	x													x											
Worcester Polytechnic Institute	8		x		x	x			x	x						x				x				x											
Yale University	1																							x											
sum of focal																																			
ASEE																																			
NSF																																			
