# Supplementary material for: Regional, institutional, and departmental factors associated with gender diversity among BS-level chemical and electrical engineering graduates
Source: PLoS One. 2019 Oct 9;14(10):e0223568. doi: 10.1371/journal.pone.0223568 (PMC6785119; doi:10.1371/journal.pone.0223568)
Supplement: S2 Table — (PDF) [file pone.0223568.s002.pdf]

S2 Table: Census Regions and Institutional Bins

	total # ChE BS	bulk % ChE female	ChE % female 2010	ChE % female 2011	ChE % female 2012	ChE % female 2013	ChE % female 2014	ChE % female 2015	ChE % female 2016	ChE slope	ChE p value for non-zero slope
**Midwest: East North Central**	5,589	30.3	30.8	28.4	26.7	31.4	31.8	31.2	31.5	0.4482	0.25
**Midwest: West North Central**	3,881	29.9	32.5	31.0	27.3	28.2	31.3	28.9	30.6	-0.2101	0.6
**Northeast: Mid-Atlantic**	4,925	36.1	37.8	33.7	34.7	35.9	38.7	36.3	35.7	0.10764	0.77
**Northeast: New England**	2,470	36.6	38.8	43.4	36.0	34.7	34.8	35.9	34.7	-1.0271	0.088
**South: East South Central**	2,345	34.5	34.3	38.4	33.8	30.8	38.6	31.7	34.9	-0.2466	0.7
**South: South Atlantic**	6,147	32.5	36.8	30.6	32.5	33.0	31.1	30.9	33.9	-0.3348	0.47
**South: West South Central**	3,920	33.5	30.8	33.8	34.8	32.0	35.7	33.3	34.0	0.33764	0.32
**West: Mountain**	3,300	29.1	30.3	30.6	29.2	25.4	29.0	29.3	30.8	-0.0452	0.91
**West: Pacific**	5,126	32.1	36.7	33.3	32.4	30.4	31.4	30.7	31.6	-0.7592	0.044
**private institutions**	6,529	40.1	42.0	38.0	38.2	42.0	40.9	40.7	38.7	-0.0664	0.862
**public institutions**	32,091	31.5	33.4	32.1	30.9	30.1	32.0	30.4	32.2	-0.2061	0.398
**HBCUs**	633	50.2	58.2	58.2	60.5	41.5	46.7	49.6	46.2	-2.393	0.079
**non-HBCUs**	37,987	32.7	34.8	11.2	32.8	11.4	31.7	11.9	32.0	-0.2837	0.91
**non-PhD granting**	3,622	34.84	36.3	32.3	33.4	35.1	34.9	33.9	37.3	0.27382	0.44
**PhD granting**	33,481	32.61	34.7	33.0	31.8	31.9	33.2	31.5	32.7	-0.2691	0.22

S2 Table: Census Regions and Institutional Bins

	total # EE BS	bulk % EE female	EE % female 2010	EE % female 2011	EE % female 2012	EE % female 2013	EE % female 2014	EE % female 2015	EE % female 2016	EE slope	EE p value for non-zero slope
**Midwest: East North Central**	6,721	12.5	8.9	11.7	12.5	11.8	13.9	13.1	15.3	0.62575	0.0054
**Midwest: West North Central**	4,270	10	6.9	6.3	10.3	11.1	11.9	11.0	11.1	0.78048	0.0255
**Northeast: Mid-Atlantic**	5,947	14.2	12.7	13.5	13.2	12.2	13.7	15.2	18.2	0.88209	0.042
**Northeast: New England**	2,256	15.6	14.4	10.7	17.0	15.1	17.1	18.8	15.9	0.94818	0.14
**South: East South Central**	2,117	13.3	13.3	12.1	11.9	12.6	12.7	14.5	15.4	0.70153	0.073
**South: South Atlantic**	7,197	12.3	10.9	12.7	10.9	10.6	14.9	13.0	12.2	0.22703	0.35
**South: West South Central**	4,267	12.6	13.3	10.8	13.7	10.5	13.0	13.6	13.5	0.43756	0.53
**West: Mountain**	3,260	10.3	12.1	9.7	10.7	9.4	11.1	8.4	10.9	0.02405	0.43
**West: Pacific**	9,582	12.3	11.7	12.2	11.4	12.2	11.4	14.7	12.3	0.27471	0.302
**private institutions**	6,491	17.9	16.9	16.5	17.9	15.3	18.0	18.7	21.4	0.85034	0.068
**public institutions**	39,731	11.7	10.4	10.8	11.3	11.1	12.4	12.8	12.5	0.41692	0.0019
**HBCUs**	673	22.0	21.9	26.3	29.7	16.1	20.8	22.6	16.5	-1.8779	0.238
**non-HBCUs**	45,549	12.4	11.2	11.4	11.9	11.6	13.0	13.5	13.7	0.45483	0.0014
**non-PhD granting**	4,841	11.34	11.3	11.8	11.4	11.6	10.4	12.1	10.9	-0.115	0.559
**PhD granting**	39,993	12.64	11.4	11.5	12.2	11.7	13.6	13.6	13.9	0.4712	0.0035
