# Supplementary material for: Regional, institutional, and departmental factors associated with gender diversity among BS-level chemical and electrical engineering graduates
Source: PLoS One. 2019 Oct 9;14(10):e0223568. doi: 10.1371/journal.pone.0223568 (PMC6785119; doi:10.1371/journal.pone.0223568)
Supplement: S3 Table — (PDF) [file pone.0223568.s003.pdf]

S3 Table: State data

State	# total ChE BS, 2010 - 2016	# total EE BS, 2010 - 2016	ChE BS bulk % female 2010 - 2016	EE BS bulk % female 2010 - 2016	per K-12 pupil expenditures ($), average of 96/97 - 11/12										
					total	instruction total	non-instruction, non-support services	support services total	pupil support	staff support	general admin	school admin	other support services	total jobs, both genders
Alabama	664	521	38.6	18.2	7,028	4,179	480	2,369	357	308	182	433	1,088	2,230,880
Arkansas	316	294	33.9	10.2	7,138	4,261	390	2,488	335	458	203	385	1,107	1,360,940
Arizona	568	848	30.3	11.4	6,202	3,504	326	2,371	487	180	103	302	1,299	2,975,165
California	4809	8374	32.7	11.8	7,864	4,769	298	2,797	375	467	68	537	1,352	18,274,870
Colorado	664	457	32.8	11.2	7,432	4,306	268	2,858	329	373	122	498	1,536	2,636,625
Connecticut	382	353	30.9	12.2	11,633	7,251	417	3,965	703	388	244	671	1,958	1,910,905
Washington, DC	107	87	63.6	24.1	13,346	6,283	465	6,598	1,054	1,281	378	929	2,955	328,035
Delaware	448	240	24.8	12.5	10,346	6,390	407	3,549	500	140	114	590	2,205	451,135
Florida	979	1016	32.3	13.0	7,159	4,241	341	2,577	343	456	75	421	1,283	9,128,080
Georgia	1054	1575	33.8	11.2	7,889	4,963	412	2,515	371	415	107	477	1,145	4,713,920
Iowa	934	1042	34.5	10.8	7,969	4,828	423	2,719	478	377	219	446	1,199	1,640,080
Idaho	131	188	30.5	9.0	6,055	3,729	287	2,039	335	249	138	351	967	751,480
Illinois	1039	1793	31.2	13.1	8,866	5,261	306	3,300	554	378	321	462	1,585	6,632,590
Indiana	1445	1564	32.2	11.4	8,151	4,859	343	2,950	366	278	153	464	1,689	3,274,430
Kansas	607	521	32.3	9.2	7,846	4,711	398	2,737	377	332	238	477	1,313	1,479,515
Kentucky	555	746	31.5	9.5	7,264	4,336	410	2,517	301	383	186	408	1,239	2,038,920
Louisiana	681	603	32.5	9.5	7,842	4,643	475	2,723	356	395	186	426	1,360	2,116,795
Massachusetts	1478	1353	41.3	19.7	11,075	6,969	345	3,761	726	620	164	495	1,756	3,531,260
Maryland	896	998	34.5	14.8	10,183	6,210	440	3,532	446	552	99	706	1,729	3,107,930
Maine	189	140	25.9	5.0	9,655	6,029	380	3,246	445	379	291	541	1,591	703,390
Michigan	1522	1456	31.9	14.8	9,033	5,214	286	3,532	651	442	181	529	1,729	4,938,335
Minnesota	1017	1123	24.1	9.9	8,661	5,600	382	2,679	255	422	251	354	1,397	2,916,930
Missouri	758	779	34.2	11.2	7,652	4,648	332	2,673	363	343	234	446	1,287	3,020,990
Mississippi	497	343	27.4	12.5	6,372	3,766	430	2,176	284	294	193	360	1,045	1,345,155
Montana	515	196	40.6	11.2	8,072	4,923	324	2,824	433	308	241	440	1,402	504,880
North Carolina	1139	1341	32.9	10.6	6,996	4,403	391	2,202	375	242	106	453	1,026	4,640,230
North Dakota	199	259	19.1	8.5	8,046	4,875	596	2,575	314	242	368	383	1,268	365,155
Nebraska	181	403	22.1	8.4	8,294	5,333	402	2,559	355	286	281	433	1,206	988,060
New Hampshire	230	199	27.0	8.5	9,377	5,940	289	3,148	658	293	332	535	1,331	738,510
New Jersey	994	1313	36.9	13.3	13,381	7,802	426	5,153	1,270	452	329	682	2,420	4,587,250
New Mexico	271	390	39.9	14.1	7,366	4,208	344	2,814	709	285	173	439	1,207	957,905
Nevada	126	205	27.0	14.6	6,873	4,134	232	2,507	290	289	112	483	1,333	1,377,920
New York	1225	1344	39.5	17.0	13,863	9,554	321	3,988	434	369	254	554	2,377	9,781,730
Ohio	1292	1451	27.7	11.1	8,850	5,031	314	3,505	536	569	238	500	1,662	5,877,985
Oklahoma	710	533	34.6	10.9	6,527	3,683	443	2,401	427	254	209	352	1,160	1,785,480
Oregon	495	619	26.5	9.7	8,149	4,783	287	3,079	538	340	127	519	1,554	1,931,995
Pennsylvania	2706	3290	34.3	13.5	10,199	6,217	389	3,592	514	377	298	432	1,971	6,408,620
Rhode Island	191	211	33.0	9.0	10,735	6,491	306	3,937	1,088	457	172	556	1,665	560,550
South Carolina	560	756	27.3	12.4	7,484	4,358	413	2,712	532	471	91	443	1,176	2,206,485
South Dakota	185	143	33.5	9.1	6,956	4,190	341	2,426	367	293	233	345	1,187	430,310
Tennessee	629	507	38.5	14.4	6,570	4,203	330	2,037	244	365	133	360	936	3,081,950
Texas	2213	2837	33.5	13.9	7,185	4,344	363	2,478	352	386	119	398	1,224	11,952,845
Utah	836	843	16.6	6.4	5,163	3,311	320	1,531	180	224	55	312	760	1,313,655
Virginia	671	844	32.8	12.0	8,589	5,258	336	2,995	413	547	125	506	1,404	4,065,245
Washington	706	1731	31.0	15.1	7,691	4,607	369	2,716	502	348	101	459	1,305	3,380,745
Wisconsin	561	457	25.8	11.6	9,363	5,739	337	3,287	422	458	242	479	1,687	3,073,910
West Virginia	293	340	31.4	11.2	8,806	5,339	503	2,964	328	299	211	478	1,647	822,340
Wyoming	189	133	23.3	6.8	10,719	6,382	343	3,994	625	591	224	548	2,006	297,310
Puerto Rico	647	605	58.6	19.2										

census data, average of 2006 - 2010					2016 earnings ratio	% of jobs ChE	% of jobs EE	% of ChE female	% of EE female	% of all workers ChE female	% of all workers EE female	% of female workers ChE	% of female workers EE
total jobs, female	total ChE, both genders	total ChE, female	total EE, both genders	total EE, female									
1,053,955	970	325	3,255	350	0.74	0.04348	0.1459	33.50515	10.75269	0.0145682	0.0156889	0.0308362	0.03320825
644,990	180	10	995	35	0.78	0.01323	0.0731	5.555556	3.517588	0.0007348	0.0025718	0.0015504	0.00542644
1,367,620	370	40	6,965	730	0.82	0.01244	0.2341	10.81081	10.48098	0.0013445	0.0245365	0.0029248	0.0533774
8,338,360	4,930	980	42,690	4,660	0.88	0.02698	0.2336	19.8783	10.91591	0.0053626	0.0254995	0.0117529	0.05588629
1,214,285	845	120	6,515	745	0.84	0.03205	0.2471	14.20118	11.43515	0.0045513	0.0282558	0.0098824	0.06135298
919,670	595	115	2,865	225	0.79	0.03114	0.1499	19.32773	7.853403	0.0060181	0.0117745	0.0125045	0.0244653
168,305	25	10	240	25		0.00762	0.0732	40	10.41667	0.0030485	0.0076211	0.0059416	0.01485399
220,305	885	155	455	4	0.82	0.19617	0.1009	17.51412	0.879121	0.0343578	0.0008867	0.070357	0.00181566
4,347,555	750	80	10,845	945	0.87	0.00822	0.1188	10.66667	8.713693	0.0008764	0.0103527	0.0018401	0.02173636
2,251,240	1,020	45	6,345	635	0.82	0.02164	0.1346	4.411765	10.00788	0.0009546	0.0134707	0.0019989	0.02820668
780,025	485	25	2,010	155	0.77	0.02957	0.1226	5.154639	7.711443	0.0015243	0.0094508	0.003205	0.01987116
341,525	240	30	1,300	85	0.76	0.03194	0.173	12.5	6.538462	0.0039921	0.011311	0.0087841	0.02488837
3,133,605	3,005	450	10,385	980	0.79	0.04531	0.1566	14.97504	9.436688	0.0067847	0.0147755	0.0143605	0.03127388
1,545,660	1,605	250	4,390	340	0.74	0.04902	0.1341	15.57632	7.744875	0.0076349	0.0103835	0.0161743	0.02199708
699,780	415	40	1,845	90	0.77	0.02805	0.1247	9.638554	4.878049	0.0027036	0.0060831	0.0057161	0.01286118
966,210	735	105	1,670	100	0.8	0.03605	0.0819	14.28571	5.988024	0.0051498	0.0049046	0.0108672	0.01034972
1,006,380	2,710	275	1,730	150	0.7	0.12802	0.0817	10.1476	8.67052	0.0129913	0.0070862	0.0273257	0.01490491
1,721,680	1,655	320	9,020	830	0.82	0.04687	0.2554	19.33535	9.201774	0.0090619	0.0235044	0.0185865	0.04820873
1,529,850	605	95	6,845	645	0.84	0.01947	0.2202	15.70248	9.422936	0.0030567	0.0207534	0.0062098	0.042161
339,765	150	4	785	70	0.84	0.02133	0.1116	2.666667	8.917197	0.0005687	0.0099518	0.0011773	0.02060248
2,365,930	2,190	455	6,680	615	0.78	0.04435	0.1353	20.77626	9.206587	0.0092136	0.0124536	0.0192313	0.02599401
1,390,500	865	125	4,465	215	0.83	0.02965	0.1531	14.45087	4.81523	0.0042853	0.0073708	0.0089896	0.01546206
1,456,945	920	50	2,860	220	0.78	0.03045	0.0947	5.434783	7.692308	0.0016551	0.0072824	0.0034318	0.01510009
650,605	365	40	1,260	35	0.75	0.02713	0.0937	10.9589	2.777778	0.0029736	0.0026019	0.0061481	0.00537961
237,560	100	15	635	35	0.73	0.01981	0.1258	15	5.511811	0.002971	0.0069323	0.0063142	0.01473312
2,231,010	1,600	160	5,955	450	0.82	0.03448	0.1283	10	7.556675	0.0034481	0.0096978	0.0071716	0.02017024
171,935	70	0	260	10	0.74	0.01917	0.0712	0	3.846154	0	0.0027386	0	0.00581615
468,815	170	20	950	30	0.78	0.01721	0.0961	11.76471	3.157895	0.0020242	0.0030363	0.0042661	0.00639911
350,435	225	25	2,345	125	0.83	0.03047	0.3175	11.11111	5.33049	0.0033852	0.016926	0.007134	0.03566995
2,170,715	3,185	455	7,230	885	0.81	0.06943	0.1576	14.28571	12.24066	0.0099188	0.0192926	0.0209608	0.04076998
451,925	235	100	1,920	140	0.82	0.02453	0.2004	42.55319	7.291667	0.0104394	0.0146152	0.0221276	0.03097859
624,845	115	20	1,160	95	0.81	0.00835	0.0842	17.3913	8.189655	0.0014515	0.0068944	0.0032008	0.01520377
4,708,575	2,020	220	11,605	815	0.89	0.02065	0.1186	10.89109	7.022835	0.0022491	0.0083319	0.0046723	0.01730885
2,826,620	3,680	580	8,145	795	0.77	0.06261	0.1386	15.76087	9.760589	0.0098673	0.013525	0.0205192	0.02812546
833,120	645	70	2,060	200	0.74	0.03612	0.1154	10.85271	9.708738	0.0039205	0.0112015	0.0084022	0.02400615
905,205	250	25	4,180	405	0.79	0.01294	0.2164	10	9.688995	0.001294	0.0209628	0.0027618	0.04474125
3,057,460	3,700	705	9,725	540	0.79	0.05773	0.1517	19.05405	5.552699	0.0110008	0.0084262	0.0230584	0.01766172
274,715	360	40	1,125	30	0.82	0.06422	0.2007	11.11111	2.666667	0.0071358	0.0053519	0.0145605	0.01092041
1,066,920	1,530	140	3,115	225	0.78	0.06934	0.1412	9.150327	7.223114	0.0063449	0.0101972	0.0131219	0.02108874
205,295	4	4	380	15	0.78		0.0883		3.947368		0.0034859		0.00730656
1,459,990	1,400	215	2,890	100	0.82	0.04543	0.0938	15.35714	3.460208	0.0069761	0.0032447	0.0147261	0.00684936
5,451,365	10,605	1,610	19,650	2,335	0.79	0.08872	0.1644	15.18152	11.88295	0.0134696	0.0195351	0.0295339	0.04283331
579,830	315	45	2,010	80	0.7	0.02398	0.153	14.28571	3.9801	0.0034256	0.0060899	0.0077609	0.01379715
1,961,445	1,330	300	7,430	755	0.8	0.03272	0.1828	22.55639	10.16151	0.0073796	0.0185721	0.0152948	0.03849203
1,574,165	790	65	5,840	635	0.77	0.02337	0.1727	8.227848	10.87329	0.0019227	0.0187828	0.0041292	0.04033885
1,470,085	855	110	4,265	325	0.78	0.02781	0.1387	12.8655	7.620164	0.0035785	0.0105729	0.0074826	0.02210757
381,835	490	80	615	0	0.72	0.05959	0.0748	16.32653	0	0.0097283	0	0.0209515	0
134,615	165	10	260	0	0.77	0.0555	0.0875	6.060606	0	0.0033635	0	0.0074286	0
