# Supplementary material for: Regional, institutional, and departmental factors associated with gender diversity among BS-level chemical and electrical engineering graduates
Source: PLoS One. 2019 Oct 9;14(10):e0223568. doi: 10.1371/journal.pone.0223568 (PMC6785119; doi:10.1371/journal.pone.0223568)
Supplement: Attachment — Submitted filename: response to reviewer comments.docx [file pone.0223568.s004.docx]

We thank the reviewers for their thorough review and helpful suggestions. A point-by-point response is provided below.

**Reviewer #1**

Comment: I could not find figure 1A. Please include it in the revised manuscript.
Response: It seems that Figures 1 and 2 were placed in incorrect order in the manuscript. This has been corrected.

Comment: the manuscript states that "On average, ChE departments have 45+/-4% higher gender diversity among BS graduates the EE departments." Based on the figure, it look like ChE departments have nearly double the gender diversity of EE departments. Please clarify this and any similar calculations in the manuscript.

Response: Thank you very much for catching this discrepancy. This had been updated, as has a similar error in the “regional variation: employment trends” section.

**Reviewer #2:**

Comment: The % of female BS Chemical Engineering (ChE) graduates is an exemplar of high gender diversity among engineering disciplines whereas BS Electrical Engineering (EE) graduates exhibit a very low female fraction, both regardless of tenure-track female faculty representation (viz., not correlated). State industry female representation has a positive association with ChE but none with EE. K12 support service funding associate positively with gender diversity in both fields. Gender trends appear stable in both fields.
While these data are of great importance to many in STEM fields, especially those in engineering and ChE and EE educators in particular, the author has not discussed the significance (beyond the statistical, that is).

Comment: The claims are very modest and entirely statistical, in my assessment.
Response: The reviewer is correct that I have been cautious in interpretation of these results. I hope that my presentation of these results will spur further analysis by those with expertise in this field. I have added a few more sentences to the discussion in response to this comment.

Comment:
- Check subject-verb agreements with criteria and data (both plural), and use of criteria where “criterion” may be the intended word for use.

Response: Done, thank you.

- P14 line 276 – “...associated significantly associated (sic) with ...”

Response: Done, thank you.

- P20 Discussion – some confusion between “instructional staff support services” mentioned in Results (p19, lines 366-69) to correlate with gender diversity at the BS level, and then the use of the term non-instructional “support services” in line 399 that seems to conflict with K-12. This could be a simple oddity of how each institutional level categorizes/labels their spending, or it may be a much deeper point to consider, as it seems to be phenomenological in functional contradiction to what actually makes a difference at different levels of instruction, how they are executed, and/or some other causal connection(s).

Response: Yes! I agree that the descriptors used by the Educational Finance Branch are very confusing. But, they are what they are. I have edited the text, Figure 5 and Table 3 in an effort to increase the clarity of how these various spending types are presented while staying true to the language used in the source documents.

- Recognizing that this is not precisely within the paper’s scope, a quick reference and correlation of EE to Physics and ChE to Chemistry at least as a parting comment in the Discussion might elucidate or at least point toward a similarity in a more robust and even causal/historical context than comparing EE with CompE to ChE with Bioengineering/Bio-related degrees. Many ChE programs and industry positions grew historically from chemistry, whereas many EE programs arose historically from Physics. The gender diversity of these two additional science disciplines should be reported/referenced at least in passing—perhaps that is a lead-in to a follow-up, causation study made as a concluding remark herein for the author’s next project.

Response: Thank you for this suggestion. This information has been added to the final paragraph of the discussion section.
